# Kosher and Trifa

**Published:** 1890-12-27

**Authors:** 


### THE DOCTOR'S ARMCHAIR.

### KOSHER AND TRIFA.

SCIENCE knows nothing of truth out of date. To the infinite intelligence time and space have no existence. As Carlyle says, to that intelligence Space is a universal "Here," and Time an everlasting "Now." The value of truth can in nowise be diminished by the fact that it was known three or four thousand years ago: to the large and reverent mind it is rather increased. The Jews of the present day claim that they are long-lived, and have great powers of endurance; and they attribute their longevity and powers of endurance to the food habits of their long line of ancestors, and to the constitutions built up and inherited as the result of those habits. Says a recent Jewish writer, addressing his fellow Jews on what he considers their dangerous readiness to adopt Gentile food habits that are injurious to health: "I will not select my text from the dicta of Rabbinic authority: I will not even adduce words taken from the Mosaic code. I will go further back still, to the time when we can scarcely speak of national law, or even of nationality itself; and thence will I draw my support in favour of the legislation which, religiously speaking, has governed our diet as Jews for thousands of years, and whose protective power may be sufficient to explain the marvellous mental and physical endurance of the Jewish people."

Moses was a practical scientist, a Bacon rather than an Aristotle; and if he had lived in our time and country, would have been President of the Royal Society. But the Jewish author, whom we have already quoted, points out that some of the most fundamental laws of the Hebrew Sanitary Code were promulgated long anterior to the birth of Moses, or even that of Abraham. To Noah seems properly to have belonged the honour of separating "clean" from "unclean" animals, and whatever was the source of Noah's knowledge, and whatever the degree of its accuracy, as tried by modern standards, there is no doubt at all that animals differ very greatly in food value and in physiological wholesomeness. It is equally certain that some of the animals classed as "unclean" by Noah, and later still by Moses, notably the omnivorous pig in its undomesticated condition, could not be eaten either with advantage or safety. Certain persons are inclined to ridicule the Jewish classification of "clean" and "unclean" beasts, on the ground that it does not accord in every particular with modern scientific knowledge. But this is truly absurd. The man of scientific instincts sees at once that the important thing is that any attempt at classification, even of the most rudimentary kind, was made at all. The separation of animals that were fit for human food from those that were unfit was a clear proof that knowledge had been acquired, and experience accumulated. Indeed, if we were disposed to rear a pyramid upon its apex instead of upon its base, according to the fashion of those who place a ton of speculative hypothesis upon a foundation of the thousandth part of a grain of doubtful facts, we might argue that because modern Britain recognised no classification of animals into clean and unclean, whilst ancient Noachism did recognise such a classification, therefore ancient Noachism was more scientific than modern Britain. The undoubted fact is that the Hebrew race for 3,000 years and more has displayed an amount of mental and physical energy and endurance which has never been surpassed, and that the Jews attribute their energy and endurance almost exclusively to obedience to their sanitary, and more particularly to their dietetic laws.

A visit to a Jewish butcher's shop would probably surprise some of our physiologists, and might be of great advantage to them. The Jewish butcher is not an uneducated man, like the average British knight of the poleaxe; indeed, he does not use the poleaxe at all. He is specially trained to kill; and to kill painlessly. But what is more, at any rate from a consumer's point of view, he is specially trained to inspect the slaughtered carcase; and to inspect it accurately. When the Jewish butcher has inspected his slaughtered animals, he divides them into two classes. The one class he calls "Kosher," and the other "Trifa." Every carcase of a slaughtered animal that is perfectly free from disease of every kind, and in all its parts, he puts aside as "Kosher," or wholesome meat; and that may be freely eaten by the Jews. Every carcase, on the other hand, that shows any indication of disease, however slight, in any of its organs, is rejected as "Trifa," and is not permitted by the Jewish law to be eaten. In ordinary cases it would have to be destroyed; but probably in these times it may be sold to the less particular and less scientific Gentiles.

No physician who has a practical grip of modern science can fail to see how very important this thorough inspection and classification of animal food is. To quote the words of the Jewish writer already referred to: "When we have the declaration upon oath of an Inspector of the Metropolitan Meat Market, that 80 per cent. of the meat sent to the London market had tubercular disease, and when in conjunction with this statement we bear in mind that the flesh and milk of cattle thus affected communicate the disease to man," is it not certain, beyond the possibility of doubt, that those persons who eat only meat which has been inspected and selected by a trained and competent inspector, have a very great advantage over those who eat indiscriminately all kinds of meat which has hardly been inspected at all? We have often in these pages pointed out the relative importance in daily life of food and physic. Where one person takes physic every day, ten thousand take food. And yet physic is placed under the strictest possible State regulations; and no man can justify himself to the law in prescribing it until he has passed through a State-regulated curriculum of training, and acquired a State-bestowed diploma of competency. But to kill animals and sell their carcases, which may be gorged in every part by diseased bacteria, requires no state qualification at all, and the killer is placed under hardly any state regulations of any kind. The public has been taught to recognise the peculiar position held by that "Doctor of Drains," called the plumber; and it is now beginning to call out for his registration. It is, at least, as necessary to have the purveying of butchers' meat placed under thoroughly scientific control as to insist that plumbers shall not fall below a certain level of intelligence and efficiency.

What a curious commentary it is on the liberality and freedom from prejudice of the scientific mind to know that the truly wonderful scientific acquirements of Moses excite no admiration at all compared with the industrious, but commonly less accurate generalisations of Aristotle? The usual habit among scientific men is to ignore Moses altogether; and even when they are compelled to admit that he was a wonderfully intelligent and practical sanitarian, as well as one of the world's greatest leaders of men, they do it unwillingly and with a grudge. Do they ever reflect how surely time brings its revenges, and that, perhaps, not a single scientist now living has established a reputation which will survive even a fiftieth part as long as that of Moses has survived already? True science knows neither race nor creed: it knows only men, and the nobleness of their character, and the greatness of their work. Socrates, it is said, "brought philosophy from the clouds; but the English have degraded her to the kitchen." One of the greatest of all the immortals shall he be who brings science from past and present, from sea and land, from sky and laboratory, and makes her the daily light and benificent guide of every humblest day labourer who delves the ground or reaps the golden grain.

